# Traces elements in feces of Eurasian otters from the mountain stream Javorinka (Slovakia)

**DOI:** 10.1007/s11356-025-36730-8

**Published:** 2025-07-15

**Authors:** Alica Furendová, Tatiana Pitoňáková

**Affiliations:** 1 Institute of High Mountain Biology, University of Žilina, Tatranská Javorina 7, 059 56 Tatranská Javorina, Slovakia; 2 Department of Microbiology and Immunology, University of Veterinary Medicine and Pharmacy in Košice, Komenského 73, 041 81 Košice, Slovakia

**Keywords:** *Lutra lutra*, Javorinka, Feces, Heavy metals, Elements, Slovakia, Mountain stream

## Abstract

The aim of this study was to evaluate the concentrations of elements and heavy metals (Hg, P, S, Cl, K, Ca, Ti, Cr, Mn, Fe, Cu, Zn, Se, Rb, Sr, Zr, Mo, Sb, Ba, and Pb) in fecal samples of the river otter (*Lutra lutra*) which inhabits the Javorinka mountain stream in the High Tatras, part of the Western Carpathians in Slovakia. Javorinka serves as a model stream with minimal human impact. As a predator, the otter is a suitable bioindicator of the environment it inhabits. Elements and heavy metals were analyzed using the energy-dispersive X-ray fluorescence (ED-XRF) technique due to its simple sample preparation requirements. Mercury was analyzed using a DMA-80 evo mercury analyzer to ensure the accurate detection of low mercury concentrations. Principal component analysis (PCA) was used to identify environmental differences in elemental and heavy metal accumulation, revealing distinct patterns influenced by water flow velocity, elevation, and prey availability. Notably, mercury showed significant seasonal variation in spring, decline, likely related to changes in the otter’s food sources. Other elements, such as sulfur, zinc, and iron, exhibited complex interactions and seasonal dependencies.

## Introduction

The river otter (*Lutra lutra*) is a well-known bioindicator of the environment it inhabits. This semi-aquatic predator is an indigenous, protected species of Slovak fauna and ranks among the largest European members of the weasel family (Mustelidae) and the order of carnivores (Carnivora) (Urban and Kadlečík 2001; Eccles et al. 2017, 2020).

This research spanned a 2-year period (September 2021 to September 2023). Aquatic ecosystems are primary reservoirs of pollutants that pose a significant risk due to their toxicity, persistence, and bioaccumulative properties (Pujari and Kapoor 2021). Pollution can stem from natural sources such as volcanic eruptions and rock weathering, as well as from anthropogenic activities linked to industry, urbanization, and the use of chemical fertilizers (Kar et al. 2008; Pujari and Kapoor 2021).

Monitoring the presence of heavy metals in the environment is crucial for ensuring environmental safety and protecting human health and ecosystems (Kar et al. 2008). Analyzing wildlife feces provides a practical method for assessing heavy metal exposure, offering insights into the daily intake of contaminants by specific species (Mason and Macdonald 1987; Mason and Ratford 1994; Delibes et al. 2009). While some heavy metals like copper, iron, manganese, nickel, and zinc are essential micronutrients for biological processes, others—such as cadmium, chromium, and lead—are harmful when they exceed certain threshold levels (Marschner 1995; Bruins et al. 2000). Exposure to elevated levels of these toxic metals has been linked to a wide range of health problems, including neurological disorders and reproductive abnormalities (Abbasi et al. 1998; Johnson 1998). Harmful elements are thought to remain in the tissues and organs of otters, but some authors (Rodríguez-Estival et al. 2020) confirmed a prey-predator cycle where heavy metals were detected in feces after consumption of contaminated prey. The Eurasian otter (*Lutra lutra*) lives a solitary life, only coming together with females during the mating season, which usually takes place in the spring. Due to this lifestyle, otters serve as suitable indicators of environmental contamination in their specific micro-region. They mark their territory with droppings and scent trails, while also constructing dens and defining home ranges along the banks (Krištofík and Danko 2012; Pitoňáková 2022).

In a previous study, we focused on monitoring mercury (Hg) levels in the environment using otters, analyzing various tissues such as guard hair, underfur, kidneys, and liver in a region of the Western Carpathians in Slovakia. We approached this study not only from the perspective of otter ecology but also considered the broader consequences of environmental pollution (Pitoňáková 2022). Subsequently, we conducted additional research on the cycling of several elements and heavy metals to gain a better understanding of their absorption, considering various influencing factors.

The main objective of this study is to analyze the content of elements and heavy metals in the feces of Eurasian otters (*Lutra lutra*) from the Javorinka mountain stream in Slovakia. The study aims to assess seasonal variations, environmental influences, and the role of otters as bioindicators of ecosystem health.

## Materials and methods

### Study area and samplings

The study area on the Javorinka River is located in the Eastern Tatra Mountains in Slovakia, specifically in the High Tatras and the Belianske Tatras. Javorinka is a Tatra stream with a characteristically steep gradient in a spruce forest. In its lower reaches, the stream flows gently with side channels and coves.

**Fig. 1:**
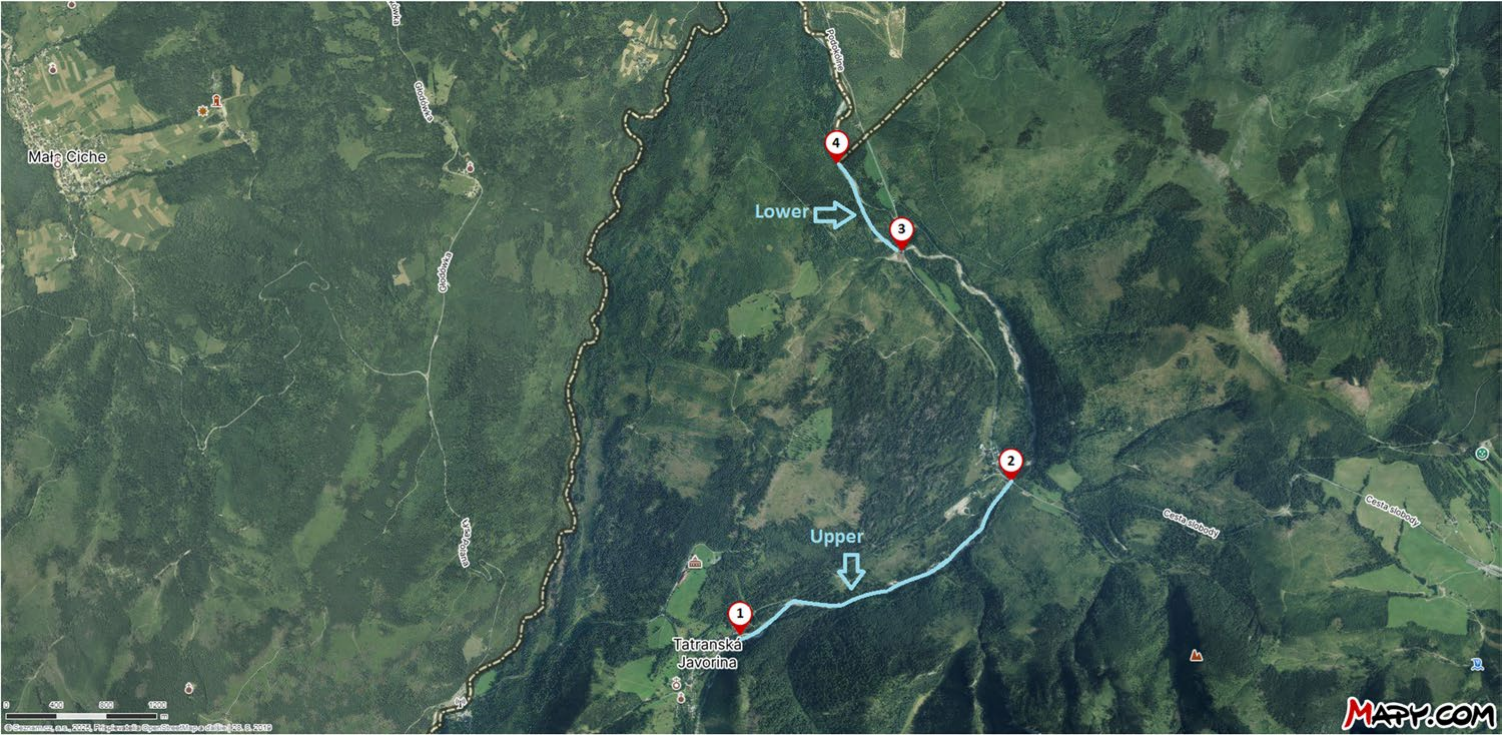
Location of sampling sites on the map. Points 1–2 mark the beginning and end of the first sampling site—upper stream. Points 3–4 mark the start and end of the second sampling site, called the lower stream (Source: https://sk.mapy.cz/ 2025)

The stream was divided into two sections for the study: the upper and lower reaches (Fig. 1). The upper stream was located at an elevation of 916 to 980 m above sea level, featuring turbulent, fast-flowing water with numerous small but deep lagoons that provide ideal habitats for fish and other aquatic organisms. This section is approximately 3 km long. The coordinates for the start of the upper section are 49°16.13000′ N, 20°8.80760′ E at 980 m a.s.l., and for the end, they are 49°16.78897′ N, 20°10.59653′ E at 916 m a.s.l. The lower stream section with an altitude ranging from 861 to 866 m above sea level is approximately 1 km long. Along this portion of the Javorinka, the banks are primarily gravelly or alluvial, with rocks and boulders. In the middle of this section, there is a stagnant stretch of the river with boulder, rock, and clay sediments deposited by stormwater. The coordinates for the start of the lower section (located approximately 200 m below the village) are 49°17.77840′ N, 20°9.87490′ E at 866 m a.s.l., and the end coordinates are 49°18.15243' N, 20°9.44945' E at 861 m a.s.l. Samples were collected monthly from both sections of the stream. The upper reach was sampled 111 times, and the lower reach was sampled 112 times. Feces were gathered using tweezers and stored in resealable new single-use plastic containers, then transported to the IHMB laboratory for analysis.

### Laboratory analysis

The collected samples were air-dried at room temperature in the laboratory for approximately 1 week. Subsequently, the samples were ground using a CryoMill (Retsch, Germany) at a frequency of 30 strokes per second for 80 s. An analytical technique also called X-ray fluorescence spectrometry (XRF) is used to detect the presence of elements. A single sample is measured in three phases and the total time to evaluate the sample and produce an arithmetic mean of the three phases is approximately 13 min (Malvern Panalytical Ltd.). We use an ED-XRF instrument from the DELTA series (BasRudice s.r.o., Czech Republic), referred to as ED-XRF spectrometry. The measurement method was standardized through the analysis of the certified reference material Beef Liver NCS ZC 71001 (China). For certain elements, the calibration factor was subsequently calculated as the ratio of the average values from three measurements of the reference material to its certified values. This calibration factor was then applied in the analysis of fecal samples. Control measurements of the reference material showed good agreement with the certified values within the uncertainty, with the relative standard deviation (RSD) being better than 10%.

The total mercury content was measured separately, directly using a DMA-80 evo mercury analyser (Milestone, Italy). The certified reference material, NCS ZC 71001 Beef Liver, was used to verify the accuracy of the measurements.

### Statistical analysis

Statistical analyses were performed using Statistica 12.0 software (StatSoft CR, Prague, Czech Republic). To examine synergistic effects, principal component analysis (PCA) was applied, a widely used technique in ecotoxicological research for reducing the dimensionality of large datasets and identifying key patterns in ecological studies. PCA maximizes the variance explained by a smaller set of components, which represent combinations of the original variables. The number of principal components (PCs) to be interpreted was determined based on the explained variance and ecological relevance, with careful attention to distinguishing meaningful patterns from random noise, as emphasized in previous studies (Bartholomew 2007; Jolicoeur 1984; Jackson 1991).

To confirm the presence of synergy, the Welch test was employed as an alternative to the standard *t*-test when variances were found to be significantly different between groups. This non-parametric approach provided a more robust analysis under conditions of unequal variances. Basic statistical operations were also performed according to the methods outlined by Furendová and Pitoňáková (2024).

Elemental analysis was performed using an XRF spectrometer (Innov-X Delta), measuring elements including P, S, Cl, K, Ca, Ti, Cr, Mn, Fe, Cu, Zn, Se, Rb, Sr, Zr, Mo, Sb, Ba, and Pb. Elements such as Co, Ni, As, and Ag were excluded from the analysis due to concentrations below the detection limit (< LOD). Mercury (Hg) concentrations were analyzed separately using the DMA 80 evo system.

## Results

Principal component analysis (PCA) revealed seven factors, with each accounting for up to approximately 5% or more of the variation or more (Table 1). Factor 1 demonstrated a synergistic relationship among S, K, Cr, Mn, and, Fe, which was associated with seasonal variations. Factor 2 revealed an antagonistic relationship between S and Sr, Fe, and Zn, influenced by altitude and season. Factor 3 represented a biogenic factor involving P, Ca, and Zn. Factors 4 and 5 emphasized the effects of altitude and flow. Factors 6 and 7 highlighted the behavior of individual elements that were not correlated with the other variables.

**Table 1:** Factor coordinates of the variables, based on correlation with their variance (%). Significant differences are marked in bold

	Factor 1	Factor 2	Factor 3	Factor 4	Factor 5	Factor 6	Factor 7
Average flow rates for 10 days	0.0656	− 0.3062	0.2463	− 0.3873	**− 0.6507**	− 0.3303	− 0.3330
Elevation	− 0.1631	0.0636	0.2926	**0.7431**	− 0.3322	0.3256	− 0.0120
Hg	0.2480	0.0428	0.2438	0.3616	**0.4660**	** − 0.6924**	− 0.1179
P	− 0.0279	− 0.3378	**− 0.7068**	0.2586	− 0.0184	0.0028	− 0.2395
S	**0.7400**	**0.5066**	− 0.1752	0.0141	0.0190	0.0411	− 0.1081
K	**0.8791**	0.3460	0.0426	− 0.0667	0.0792	0.1526	− 0.0866
Ca	− 0.3145	0.3083	**− 0.6333**	− 0.1249	− 0.1363	− 0.2311	0.3704
Cr	**0.9062**	0.0649	− 0.0770	− 0.0940	− 0.0063	0.1327	− 0.0984
Mn	**0.8942**	0.0503	− 0.1294	− 0.0928	− 0.0786	0.0241	− 0.0147
Fe	**0.7537**	** − 0.4719**	0.1151	0.1474	− 0.0308	− 0.0432	0.1185
Zn	0.4263	** − 0.4535**	**− 0.4085**	0.3358	− 0.1967	− 0.1353	0.0179
Rb	0.4944	− 0.3871	0.2235	− 0.0766	− 0.0460	− 0.0251	**0.6166**
Sr	− 0.0995	**− 0.6521**	− 0.0673	− 0.2507	**0.4855**	0.2989	− 0.1584
Eigenvalue	4.14	1.70	1.40	1.14	1.06	0.90	0.77
% Total-variance	31.83	13.04	10.74	8.78	8.16	6.93	5.91

**Fig. 2:**
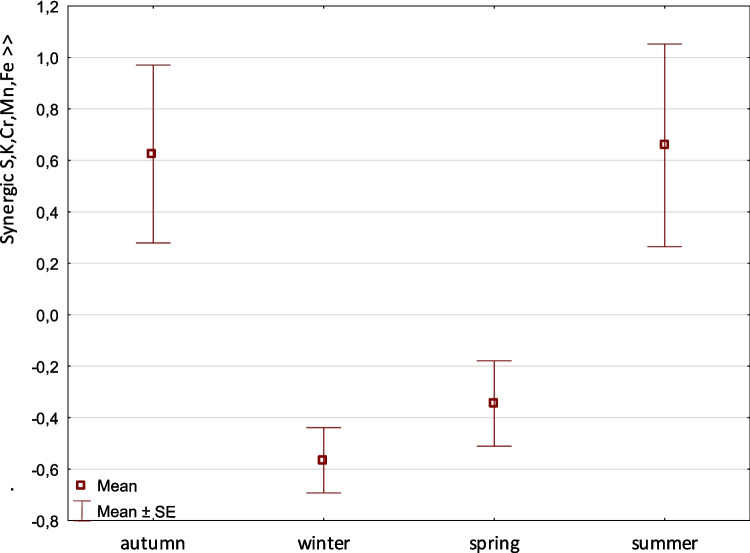
Factor 1: Comparison of means (± SE) of factor coordinates of samples among seasons. The coordinates indicate a synergic accumulation of S, K, Cr, Mn, and Fe in the feces of the European otter (F–Welch = 5.7, *p* = 0.001). Amounts of the element in autumn and summer significantly differed from the amounts in winter and spring (LSD test at *p* < 0.05)

Factor 1 showed a synergistic relationship between the elements S, K, Cr, Mn, and Fe and their abundance, with a variance of 31.83%. This factor is not related to flows or elevation but is associated with seasonal variation. It is a unipolar phenomenon. In autumn and summer, there are significantly more S-, K-, Cr-, Mn-, and Fe-based elements, either singly or combined, in the diet of river otters. The synergistic effect is demonstrated when these elements occur together. This effect begins in summer with a continuation in autumn and begins to decrease in early winter, dropping sharply in spring (Fig. 2).

**Fig. 3:**
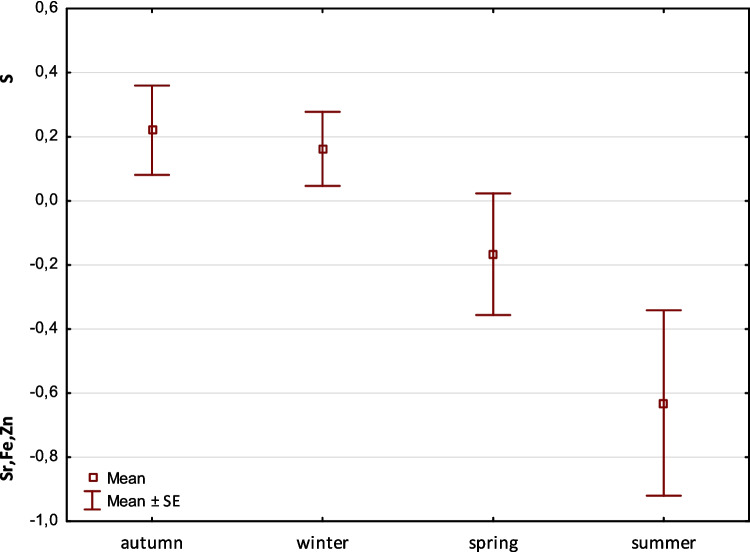
Factor 2: Seasonal comparison of mean (±SE) factor scores in otter fecal samples. The scores indicate an antagonistic pattern of accumulation—higher sulfur (S) is associated with lower levels of Sr, Fe, and Zn. Significant differences were observed in summer compared to winter, autumn, and spring (Welch’s F = 3.02, p = 0.03; LSD test* p*<0.05)

Factor 2 shows an antagonistic relationship between the elements S versus Sr, Fe, and, Zn with a scatter of 13.04%. The effect shows a decreasing tendency of Sr, Fe, and Zn when S abundance is high. This trend depends on the elevation and only partially depends on the flow rate. It shows little effect in autumn and is maintained in winter. Conversely, when spring and summer arrive, the proportion of S in the diet begins to decrease and Sr and Fe begin to rise. Since it is flow related, the effect in the otter diet is related to spring and partly to summer. This is when most metals are present in the environment (Fig. 3).

**Fig. 4:**
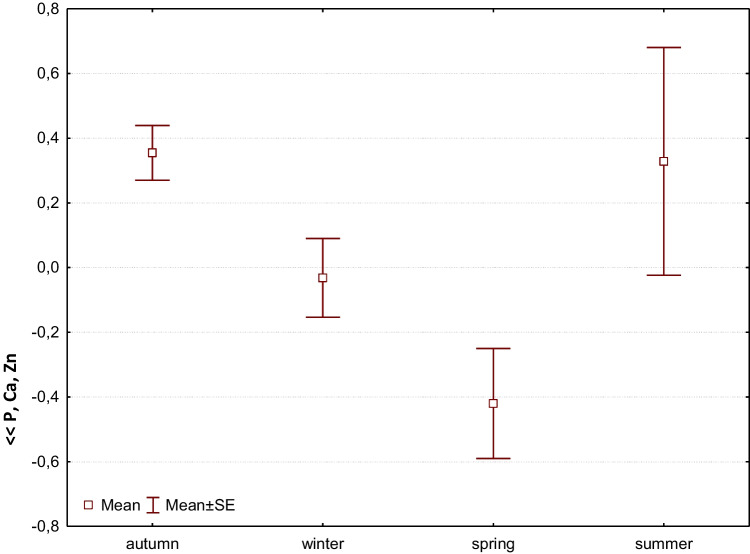
Factor 3: Comparison of means (± SE) of factor coordinates of samples between seasons. Coordinates indicate synergistic accumulation of P, Ca, and Zn in otter feces (F-Welch = 6.4, *p* = 0.0006). The amounts of biogenic elements in spring were significantly different from those in autumn, winter, and also summer (LSD test at *p* < 0.05)

Factor 3 can be called a biogenic factor, as it shows the relationship between biogenic P, Ca, and Zn, with a scatter of 10.74%. It is not related to flow rates or elevation, but shows the period when the otter receives the most nutrients into its body (Fig. 4).

Factor 4 shows the change in altitude alone, with no effect on other variables, is irrelevant.

**Fig. 5:**
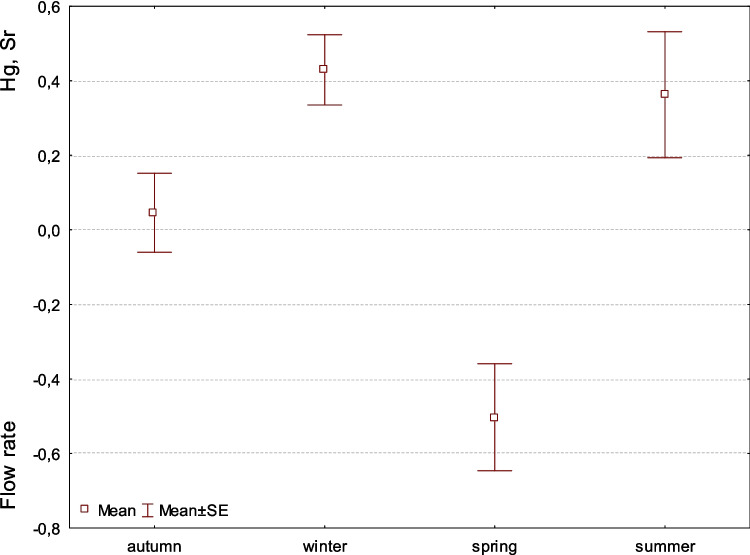
Factor 5: Comparison of means (± SE) of factor coordinates of samples between seasons. Coordinates indicate antagonistic accumulation of Hg and Sr with flow rate in feces sample of the European otter (F-Welch = 10.5, *p* < 0.01). Amounts of the element in autumn significantly differed from the amounts in winter and spring, winter also differed with spring and did not differ with summer, spring highly differed with other three seasons, and summer differed only with spring (LSD test at *p* < 0.05)

Factor 5 shows the antagonistic effect between flow rates and Hg with Sr. The lower the flow rate, the more Hg and Sr are in the environment; it is a bipolar factor. And since flows are strongest in spring, Hg and Sr levels are lowest. In winter when the flow is weakest (frost, snow), the contamination is higher (Fig. 5).

**Fig. 6:**
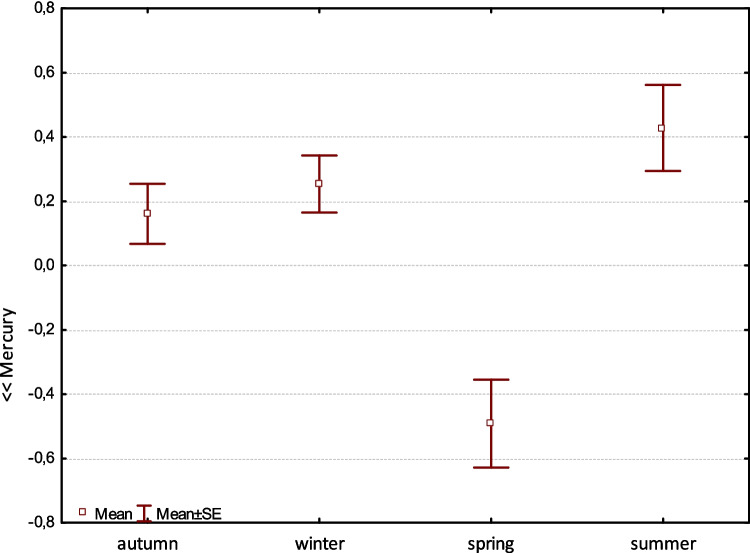
Factor 6: Comparison of means (± SE) of factor coordinates of samples between seasons (F-Welch = 9.1, *p* < 0.001). The mercury factor is independent of the previous factor. Hg abundance is significantly different in spring from other seasons

Factor 6 shows Hg alone with a variance of 6.93%, unrelated to flows and elevation. It may be a different form of Hg that enters the environment from different sources and has its own cycle regardless of environmental factors (Fig. 6).

**Fig. 7:**
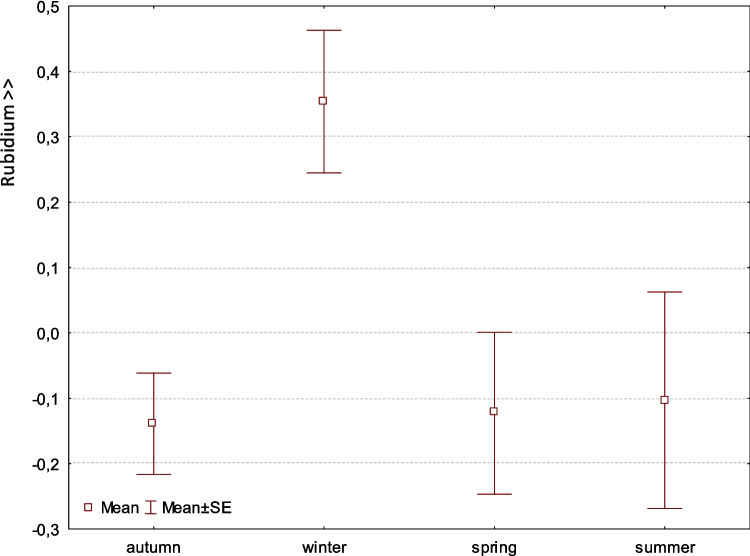
Factor 7: Comparison of means (± SE) of factor coordinates of samples between seasons (F-Welch 4.9, *p* = 0.003). The rubidium factor is independent of the previous factors. Rb abundance is significantly different in winter from other seasons

Factor 7 is the Rb factor with a variance of 5.91%. It is not related to flow rates or elevation. Its form can vary and can come from different sources (Fig. 7).

## Discussion

Principal component analysis (PCA) showed that factor 1 reflected a synergistic accumulation of S, K, Cr, Mn, and Fe in river otter feces. These elements showed a significant increase during the summer and autumn seasons, coinciding with periods of concentrated water (Fig. 2). It is hypothesized that seasonal changes in feeding habits contribute to this variation. Despite the higher diversity of fish during summer, river otters increase their consumption of amphibians, birds, and other invertebrates during this period, suggesting a seasonal dietary shift that may explain the observed variations in elemental accumulation (Hájková 2001; Pitoňáková 2022; Furendová and Pitoňáková 2024). This shift in prey composition is influenced by seasonal dynamics, prey abundance, and activity (Roche et al. 1995). For instance, brown trout (*Salmo trutta fario*) and Alpine bullheads (*Cottus poecilopus*) are dominant species in Javorinka, particularly during summer and autumn, coinciding with the migration of adult female salmonids for spawning and overwintering (Jonsson 1985; Janiga 2018). These findings suggest that otters respond to the increased availability of certain species, such as fish, which are abundant in their habitats (Brzeziński et al. 2006).

Factor 2 demonstrates an antagonistic correlation between S and elements such Fe, Zn, and Sr (Fig. 3). During spring and summer, S levels decrease while Fe, Zn, and Sr levels increase. This trend appears to be unaffected by elevation or flow rates. It is speculated that otters acquire more Fe, Zn, and Sr during summer, possibly through synergistic interactions with other food sources or internal metabolic processes. Conversely, sulfur intake increases in winter, autumn, and partially in spring. Potential sources of these elements in spring and summer include phyto- and zooplankton, carp, other fish, granitic soils, and weathering processes (Anke et al. 2005; Furendová and Pitoňáková 2024; Janiga et al. 2024). While Zn and Fe are considered essential for otters, the biological significance of Sr remains unspecified (Esposito et al. 2020). Elevated exposure to these elements may interfere with physiological processes and serve as an indicator of pollution. Sulfur may play a crucial role in this antagonistic relationship by affecting pH levels and thereby mobilizing metals (Brand et al. 2020).

Factor 3 reflects the combined synergistic effect of increasing or decreasing phosphorus, calcium, and zinc (Fig. 4). Compared to the autumn and winter seasons, there are significant fluctuations in biogenic elements in the summer months. This may be due to the change in diet during the summer months, as more options than just fish are available. In spring, intake decreases the most. In the autumn spawning season, female trout that reach a size of more than 15 cm migrate to the Javorinka River (Dyková 1964). These may represent a source of biogens for otters, the intake of which is highest in autumn ((Furendová and Pitoňáková 2024).

Factor 5 illustrates an antagonistic relationship, with an increase in Hg and Sr levels accompanied by a decrease in water flow (Fig. 5). This association implies that lower water flow correlates with higher concentrations of Hg and Sr in the environment. The study indicates no significant seasonal variation across summer, winter, or autumn. With water flows peaking during spring ice melt, Hg and Sr levels reach their minimum. This observation aligns with the findings of Douglas et al. (2017), who confirmed elevated Hg concentrations in snowpack and runoff from snowmelt. The leaching of strontium from glacial bedrock may be influenced by the degree of contamination and the amount of strontium present in snow layers, subsequently released into meltwater (Arendt et al. 2016).

Factor 6 represents the mercury phenomenon, differing in spring from other seasons and independent of water or elevation (Fig. 6).With a variance of 6.93% in otter feces, mercury enters otters mainly through food, especially in spring (Furendová and Pitoňáková 2024). Studies in northeast Scotland (Jenkins 1980; Jenkins and Harper 1980; Weber 1990) showed that otters mainly consumed amphibians in late winter and spring when available, with almost 30% of feces containing amphibians in spring (Weber 1990). Amphibians were scarce in summer. Otters occasionally consumed mammals and birds, particularly in late winter and early spring, in amounts not exceeding 4% year-round (Chanin 1981; Adrian and Delibes 1987; Weber 1990; Skarén 1992). Our findings suggest that otters may heavily rely on amphibians in spring, supported by studies indicating their importance as prey during this season (Weber 1990). This contrasts with some previous studies that suggested amphibians are not a primary otter food source (Chanin 1981; Adrian and Delibes 1987; Mason and Macdonald 1987). However, studies from Poland (Brzeziński et al. 2006) reported significant consumption of frogs in autumn and winter, suggesting variability in otter diet across regions and seasons. Overall, the proportion of prey in otter diets seems to depend on habitat and prey availability (Weber 1990; Jedrzejewska and Jedrzejewski 1998; Poledník et al. 2004; Brzeziński et al. 2006; Mason and Macdonald 2009).

Factor 7 shows a seasonal variation in rubidium content, with higher values recorded during the winter months compared to other seasons (Fig. 7). This pattern is not associated with elevation or water flow rates. We hypothesize that the presence of rubidium in the otter’s feces may originate from the food it prefers in winter (Furendová and Pitoňáková 2024). It is necessary to investigate the diet of fish and amphibians in our study site, the Javorinka River, in more detail in order to pinpoint the source of this element. Rubidium occurs in elevated concentrations in juvenile fish, which may be one possible source (Kapitola and Vilimovská 1984). In addition, rubidium is found in high concentrations in plant populations on soils with high granite content (Anke et al. 2005).

## Conclusion

This study highlights the significant impact of environmental factors such as season, altitude, and water flow on the accumulation of heavy metals in the feces of Eurasian otters in the Javorinka mountain stream. The findings demonstrate distinct seasonal variations in the concentrations of various elements, reflecting changes in otter diet and environmental conditions. The research underscores the importance of ongoing monitoring to track heavy metal contamination in aquatic ecosystems. The presence of heavy metals in otter feces serves as a critical indicator of ecosystem health and potential pollution sources. It also points to the need for comprehensive studies that consider long-term trends and a wider geographical scope to fully understand the ecological impact of these contaminants.

## Data Availability

The datasets used and/or analyzed during the current study are available from the corresponding author on reasonable request. The authors declare to cite any publicly available data on which the conclusions of the paper rely in the manuscript.
